# Availability Issues in Wireless Visual Sensor Networks

**DOI:** 10.3390/s140202795

**Published:** 2014-02-12

**Authors:** Daniel G. Costa, Ivanovitch Silva, Luiz Affonso Guedes, Francisco Vasques, Paulo Portugal

**Affiliations:** 1 DTEC-UEFS, State University of Feira de Santana, Feira de Santana 44036-900, Brazil; 2 IMD-UFRN, Federal University of Rio Grande do Norte, Natal 59072-970, Brazil; E-Mail: ivan@imd.ufrn.br; 3 DCA-CT-UFRN, Federal University of Rio Grande do Norte, Natal 59072-970, Brazil; E-Mail: affonso@dca.ufrn.br; 4 IDMEC-FEUP-UP, University of Porto, Porto 4200-465, Portugal; E-Mail: vasques@fe.up.pt; 5 INESC TEC-FEUP-UP, University of Porto, Porto 4200-465, Portugal; E-Mail: pportugal@fe.up.pt

**Keywords:** wireless visual sensor networks, availability, directional coverage, coverage metrics

## Abstract

Wireless visual sensor networks have been considered for a large set of monitoring applications related with surveillance, tracking and multipurpose visual monitoring. When sensors are deployed over a monitored field, permanent faults may happen during the network lifetime, reducing the monitoring quality or rendering parts or the entire network unavailable. In a different way from scalar sensor networks, camera-enabled sensors collect information following a directional sensing model, which changes the notions of vicinity and redundancy. Moreover, visual source nodes may have different relevancies for the applications, according to the monitoring requirements and cameras' poses. In this paper we discuss the most relevant availability issues related to wireless visual sensor networks, addressing availability evaluation and enhancement. Such discussions are valuable when designing, deploying and managing wireless visual sensor networks, bringing significant contributions to these networks.

## 1. Introduction

Wireless sensors networks (WSNs) have fostered the development of a large set of unattended applications for Internet-based monitoring networks, and have become a relevant research topic in the last years [1]. When source nodes were equipped with low-cost and low-power cameras, a new research area was opened since new challenges demanded additional investigation efforts to address a whole new group of communication requirements [2]. In fact, transmissions of visual data usually require more communication bandwidth than transmissions of scalar data, besides the additional processing and energy costs for data sensing and coding at source nodes, turning visual monitoring by wireless sensor networks into a challenging task [3,4].

Visual surveillance applications may be composed of hundreds or thousands of sensors for monitoring static or mobile targets. In general terms, different types of sensors may be employed, making up from homogeneous single-tier monitoring networks to complex multimodal heterogeneous wireless multimedia sensor networks for content-rich monitoring [5,6]. Whatever the case, a subset or all camera-enabled sensors will retrieve visual information from the monitored field, which will be delivered through *ad hoc* wireless links to the sink of the network [1–3].

There are some crucial issues that must be properly addressed when performing visual monitoring. The opposition between the stringent requirements of visual data transmission and the resource constrained nature of wireless sensors imposes the need for energy efficiency over the network [7]. In such a context, different optimizations have been proposed aiming at achieving energy efficiency with reduced impact on the monitoring quality [2,8]. From a different perspective, reliable communications may be required for visual monitoring applications, since some packets may be highly necessary for reconstruction of the original data [8]. Moreover, some visual sensors may retrieve critical information for the application, depending on the application requirements and cameras' poses [9], also requiring reliable transmissions. Similarly, many works have addressed the topic of reliability in wireless visual sensor networks (WVSNs) through packet retransmission, correction codes or packet-level redundancy, acting on different aspects of sensing, coding and transmission [10–12]. In fact, most research efforts in the wireless visual sensor network field have been focused on mechanisms to assure energy efficiency and reliability in these networks, making relevant contributions.

A failure may impair the monitoring capability of wireless visual sensor networks, where the impact will vary according to the nature of the monitoring applications. While failures in source nodes may reduce the amount of visual information that reach the sink, potentially impacting the monitoring quality of surveillance applications, failures in critical monitoring applications for industrial control or medical assistance may result in economic losses or put people in danger [13]. In short, a system failure will be caused by transient or permanent faults [14], where transient faults usually affect communication links due to noise or interferences and permanent faults result from hardware malfunctions, sensor damage or energy depletion [13,14]. A transient fault will directly impact packet transmission, requiring proper mechanisms to assure some level of reliability. On the other hand, permanent faults may make part or the entire network unavailable. Although reliability in wireless visual sensor networks has been broadly addressed in some recent works, availability is still an open research topic.

An efficient way to enhance availability is by deploying redundant sensors, which will be used to compensate permanent failures in active nodes. Usually, redundancy may be obtained with massive deployment or deterministic positioning of sensor nodes [9,14]. In scalar wireless sensor networks, redundancy has been widely considered as a practical mechanism to extend the network lifetime when sensors run out of energy [15,16]. Proper investigations have been also conducted to address redundancy in wireless visual sensor networks, focusing on network lifetime extension [17] and localization of visual sensors exploiting overlapped areas [18,19]. In a different way, sensors embedded with adjustable cameras may be deployed, allowing horizontal and vertical movement for optimal visualization of the monitored field and dynamic monitoring of mobile targets. Such adjustable cameras may also be exploited to enhance the level of availability of wireless visual sensor networks.

In WVSNs, redundancy depends on the nature of the application, since each camera-enabled sensor probably has a unique view of the monitored field [9,20]. For surveillance applications, for example, distinct viewing could provide the same information for the application processing, resulting in viewing redundancy. However, visual data transmitted from different source nodes may be processed by applications as completely different information [9]. Moreover, source nodes may have different relevancies for the applications, where the importance of each source node is a direct function of the expected targets to be monitored, instead of the deployed network characteristics [21], resulting in source nodes with different priorities. In short, wireless visual sensor networks may have different notions of redundancy and vicinity, according to the application requirements and network configurations, requiring a particular perception of availability for each type of visual monitoring application. This aspect increases the possibilities of permanent failures that can affect availability.

In this paper we discuss the main availability issues of wireless visual sensor networks, according to the nature of the desired monitoring functions. These issues are highly relevant when evaluating the availability of visual monitoring applications, potentially helping in planning and management of these networks. We also discuss practical approaches to enhance availability in WVSNs.

The remainder of this paper is organized as follows: Section 2 describes the fundamentals of directional sensing in wireless visual sensor networks, classifying visual monitoring into three different categories. Node failures in wireless visual sensor networks are discussed in Section 3. Section 4 addresses availability evaluation. Some practical approaches to enhance the level of availability of visual sensor networks are discussed and proposed in Section 5. Finally, conclusions are presented and references listed.

## 2. Redundancy of Visual Sensors

The concept of availability in wireless visual sensor networks can be thought of as the capability of the network to provide the required information for the application. In such a way, availability in WVSNs will be related with the coverage quality of the network, which is strongly associated to redundancy. In short, redundancy is achieved when two or more sensors are retrieving equivalent information, but different applications may have different notions of equivalence according to their monitoring requirements. Thus, identifying redundancy among sensors is a complex task, bringing many challenges to availability estimation, evaluation and enhancing.

Redundancy in wireless visual sensor networks will depend on the nature of the monitoring applications. We classify redundancy in three different categories, according to the importance of the retrieved visual information, where redundancy may be defined considering FoV overlapping, sensing similarity or sensing relevance. The classification of redundancy in three different categories originates primarily from the way visual information is gathered by the cameras. In fact, visual sensors collect data following a directional sensing model [9], making characteristics such as resolution and orientation relevant parameters when defining redundancy.

**Figure 1.**
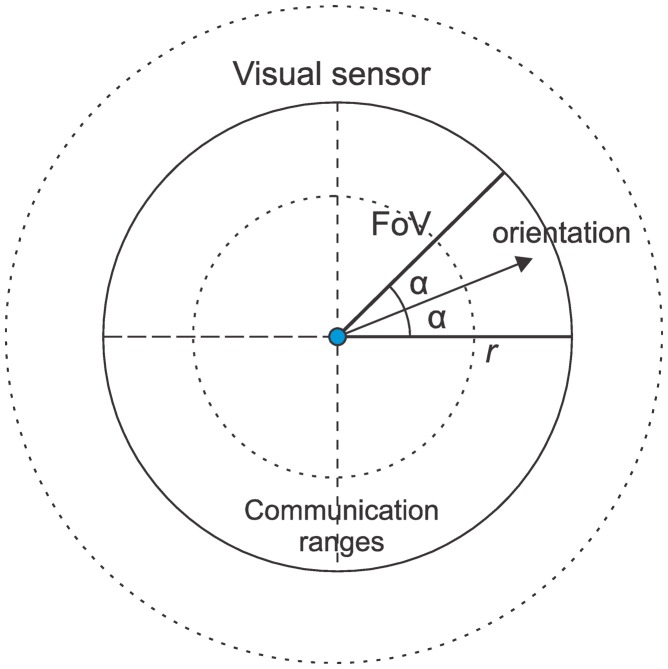
Directional sensing model. The FoV is a sector of the circumference.

In scalar wireless sensor networks, the sensing range of the nodes can be approximated to the radius of a circumference [22]. In such way, neighboring nodes are likely to collect similar data and sensing and connectivity scopes are equivalent, making availability evaluation and enhancement in traditional wireless sensor networks easier. On the other hand, camera-enabled sensors collect data in a different way, following a directional sensing model. Thus, for visual sensor networks, the concept of vicinity is valid only for communication, which is omnidirectional, since the sensing range in wireless sensor networks is replaced in WVSNs by the Field of View (FoV) [9,20]. This particularity demands a new understating of sensing redundancy. Figure 1 presents a 2D representation of a typical camera's field of view, where the two dashed circles are examples of wireless communication ranges.

As visual monitoring depends on the sensors' poses, resolution and depth of view [9], two camera-enabled sensors can collect visual data from the same object or scene, even when they are many hops away from each other. In a different way, a very close object may be not viewed by a particular visual sensor, what would not be true for a scalar sensor network.

When evaluating the availability level of wireless visual sensor networks, we will usually be concerned with the coverage level of the network, as will be discussed in Section 4. In other words, we want to know how well an area of interest is covered by source nodes, which may indicate if the deployed network can provide valuable information for the monitoring applications. But such an evaluation will need to regard the way redundancy is implemented in the network, which will be a direct function of the characteristics of the defined categories of redundancy. In fact, visual coverage is strongly related with redundancy, since redundant nodes can be exploited to keep a minimum level of coverage quality (required by the application) and connectivity of the nodes while extending the network lifetime.

**Figure 2.**
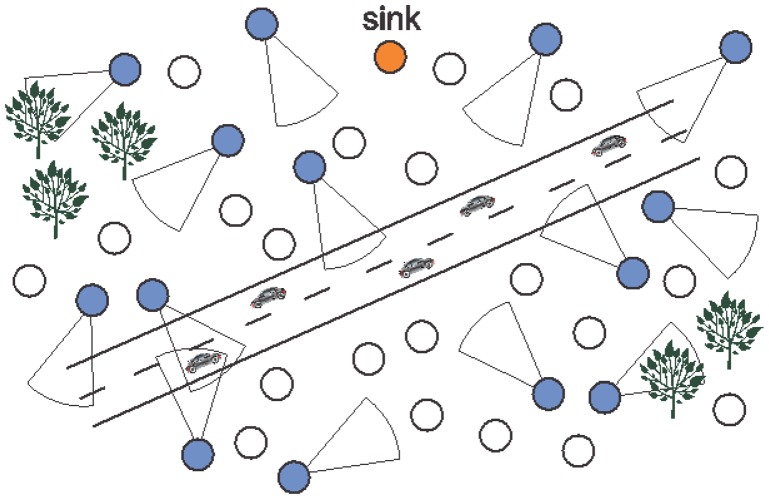
A typical wireless visual sensor network.

Figure 2 presents a generic wireless visual sensor network deployed for street monitoring, where camera-enabled sensors (blue circles) will retrieve visual information from cars and their vicinities. The remaining sensors (white circles) are employed to create *ad hoc* multihop transmission paths.

We can roughly expect that visual sensor networks are composed of three types of sensors: visual source nodes, sensor nodes and inactive nodes. Visual sources are camera-enabled sensors that collect information according to their FoV. Sensor nodes are source scalar sensors, intermediate relaying nodes that make up *ad hoc* transmission paths or even visual sensors that are not retrieving visual information (acting only as relay nodes). Finally, inactive nodes are any kind of sensor that is not being currently used, mainly because it is assumed a redundant node for a current active sensor.

Following a directional sensing model, every visual sensor has a unique view of the monitored field. However, the interpretation of the retrieved information relies on the particular monitoring requirements of the application of concern. For a visual surveillance application, for example, distinct viewing could provide the same information for the application processing, resulting in visual redundancy. On the other hand, some monitoring applications will strictly process the retrieved information, and visual data resulting from different perspectives of the same target will be processed as different information. Finally, visual sensors may have different sensing relevancies for the application and redundancies will be associated with monitoring priorities. Such visual monitoring paradigms are significant not only when planning and deploying the network, but also when identifying redundant nodes. The next sections describe each of the proposed visual sensor redundancy categories.

### 2.1. Redundancy Based on FoV Overlapping

When viewing targets or scenes, the FoV of visual sensors may overlap. In other words, when the FoV of two or more cameras intersects, the same object or scene is viewed by more than one visual sensor, often from different directions and perspectives. In such a way, applications may define redundancy based on FoV overlapping.

**Figure 3.**
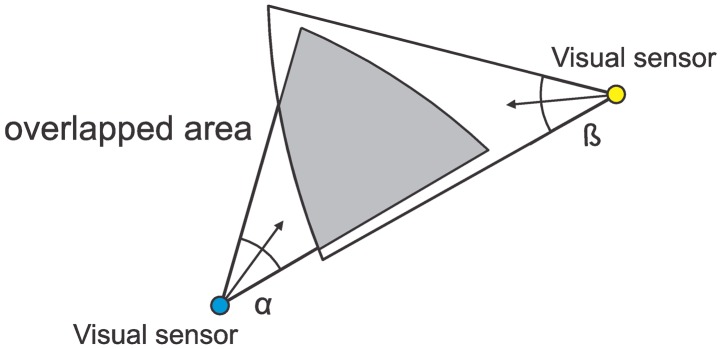
Redundancy based on FoV overlapping.

Since sensors with overlapped sensing areas view the same target, but from different perspectives, hence they retrieve different information. However, for many monitoring applications, two visual sensors may be assumed as redundant if they have some minimum level of FoV overlap (e.g., 30%) whatever are the cameras' orientations. Figure 3 presents an example of visual sensing overlapping where both sensors may be assumed as redundant.

For some applications, information transmitted from sensors with overlapped sensing coverage may provide equivalent results, as when visual sensors are used for intrusion detection and general-purpose surveillance. In Figure 2, for example, two different sensors can view the same car since they have overlapped FoV and thus they may be equivalent, depending on the application monitoring requirements.

### 2.2. Redundancy Based on Sensing Similarity

As stated before, FoV overlap may be exploited to indicate that different sensors may provide information that is somehow related, and thus those visual sensors may be assumed as redundant. However, some monitoring applications may require high similarity between visual sensors when defining redundancy, where visual sensors are said to have similarity when they have very close perspectives of the same target or scene. For example, two sensors may not be considered redundant unless they have high FoV overlap (e.g., 90%) and they also have very similar orientation (e.g., less than 10% angle difference between the cameras' orientations). This is, in fact, a particular case of visual monitoring based on FoV overlapping.

**Figure 4.**
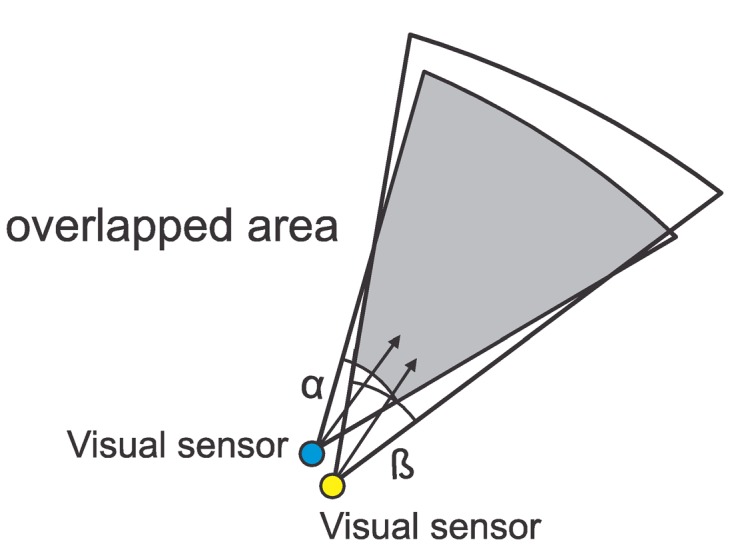
Redundancy based on sensing similarity.

If a visual sensor network is deployed for facial recognition and people are walking in one direction, for example, visual data of the people's backs are irrelevant. In such cases, sensors with high FoV overlap and those that are viewing people's faces may be assumed as redundant nodes. Figure 4 presents an example of visual monitoring based on similarity.

Applications may define a minimum level of FoV overlap and maximum acceptable angle between the cameras' orientations as thresholds when defining redundancy. This visual monitoring paradigm may typically require deterministic deployment of visual sensors to achieve optimized coverage. Other possibility is the use of PTZ cameras [23] to adjust the FoV in order to try to enhance the coverage similarity of the deployed camera-enabled sensors.

### 2.3. Redundancy Based on Sensing Relevance

Regardless of whether visual monitoring is based on overlapping or similarity, visual source nodes may have different relevancies for the applications. The sensing relevance is defined as the potential of source nodes to retrieve significant information for the monitoring application [21]. Since the sensing relevance is not associated to the network topology and sensor positioning, the same exact sensor network may have source nodes with different relevancies depending on the considered application requirements. Source nodes with different sensing relevancies will be assigned to a priority level that may be exploited in different ways, as in visual data transmission [24], error control [25] and packet routing [26], just to cite a few.

The work in [21] proposes the concept of sensing relevance in wireless visual sensor networks, defining five different groups of relevance related to the overall significance of the source nodes for the applications: irrelevant, low relevance, medium relevance, high relevance and maximum relevance groups. Each camera-enabled source node is associated to a unique group of relevance, according to its potential to provide significant visual data. In such way, the notions of redundancy and coverage quality are changed considerably, since the concept of redundant nodes is only valid within the same relevance group.

The sensing relevance may be established according to the monitoring of regions of interest. Applications may define regions where relevant data are more likely to be retrieved and sensors that can view those regions will be assigned to a particular relevance level. Using the (x,y) positions of the visual source nodes and the cameras' poses, we can identify if the FoV of the sensors are (partially) inside the regions by trigonometry. Simplifying the FoV to a triangular region, we can check if the vertices of the FoV triangle are inside the area of any region of interest, but 3D computing is also feasible [27–29]. Additionally, regions of interest may be too small just to comply with the dimensions of a specific target.

**Figure 5.**
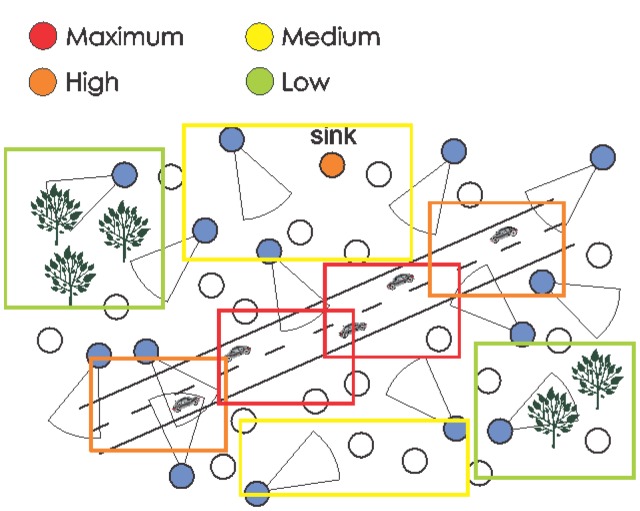
Sensing relevance according to the monitoring of areas of interest.

Figure 5 presents the same monitoring scenario described initially in Figure 2, but now assuming different relevancies according to the monitoring of areas of interest.

For the considered application, sensors that can view specific parts of the street will receive maximum relevance, while visual monitoring over the remaining parts will have high relevance. For surrounding regions, lower relevance will be assigned to the visual source nodes. One should note that the exactly same network could define other areas of interest depending on the monitoring requirements.

All sensors that belong to the same group of relevance and thus can view regions of interest with the same relevance are equivalent for the monitoring application. A redundant inactive node may replace a faulty visual sensor only if they can view a region of interest with same relevance for the application. A redundant node can replace a faulty node that was viewing a different region of interest, when those regions have same relevance. In doing so, availability evaluation and enhancement become more complex, requiring proper analyses of the current monitoring requirements of the applications.

## 3. Node Failures

Traditionally, availability is assumed as a characteristic of the network structure [30–32]. However, we believe that in wireless visual sensor networks it is also a characteristic of the applications. In other words, a network is said to be available as long as source nodes can provide relevant information for the current application. This capability of the network will be strongly associated with the use of redundancy to compensate failures, but what are node failures for wireless visual sensor networks?

Generally, a failure in a sensor node may interrupt or compromise data transmission, but the impact of such a failure may depend on the role of that sensor has for the monitoring functions of the application and how redundancy was defined. Thus, we need a different perspective of node failures.

When addressing availability, we are most concerned with permanent node failures [14]. In a generic way, a permanent failure is a condition where a sensor node is not operating as expected, which may be reflected in the way sensors produce and relay data packets. We classify node failures in two distinct groups: hardware failures and coverage failures. A hardware failure manifests when sensors run out of energy, when sensors are damaged, when they are disconnected or even when a faulty condition arises due to problems in the manufacturing process. Thus, a sensor that had a hardware failure is assumed as a faulty node for any type of application and visual monitoring paradigm. On the other hand, coverage failures may diminish the monitoring quality of the applications, when fewer visual data or lower quality images or videos are retrieved from the monitored field. This kind of failures depends strongly on the way visual information is gathered and processed by the applications and how redundancy is defined. Understanding these two different classes of failures is crucial when addressing availability in wireless visual sensor networks.

In general words, a node failure may inactivate a node for relaying functions or for sensing functions, or only compromise the quality of the retrieved data. In [33,34], a node failure is classified in *hard* or *soft*. A hard failure is the result of significant problems in some module, like communication and energy, while a soft failure does not inactivate a sensor node for the application, but the transmitted or sensed information is not correct or precise. We extend that concept considering that a visual sensor node may have a hardware or coverage failure, which may be in turn hard or soft. We could be most concerned with coverage failures since they require proper treatment due to the directional sensing nature of visual sensors, which is more challenging than scalar sensor monitoring. Additionally, hardware failures (such as energy depletion and communication interruption) in wireless sensor networks are frequently equivalent for WVSNs. In wireless visual sensor networks, we can expect hard coverage failures when sensors cannot view the intended target or scene, while soft failures may result when the camera's lens is not severely harmed (it may be cracked) or not clean.

### 3.1. Hardware Failures

Sensors nodes will typically be tiny battery-operated devices with constrained processing and memory resources that are expected to be cheap enough to allow massive deployment [1,2]. Such devices may act as source sensors, retrieving information from the monitored field, or relaying nodes, participating in *ad hoc* transmission paths, but both functionalities may be performed by a single node. Whatever the case, sensor nodes will operate until they become faulty. Wireless sensor networks are expected to face a lot of failure conditions, especially when camera-enabled sensors are deployed.

Some initial hardware failures may result from the deployment mechanism. If sensors are airdropped, they may be harmed during the fall on when they hit the ground. Considering that cameras' lens are typically fragile, they may not resist an airdrop. Moreover, wind can blow sensors away from the monitored area, making them unavailable. An additional difficulty is that visual sensors should not land with their cameras directed to the ground or to the sky (unless required by the application), since they would be useless for sensing functions [9], affecting the network availability. Visual sensors may also be accidentally deployed in dark regions or areas with high occlusion, making the retrieved information useless.

We can expect that sensors may be deployed in hazard or dangerous areas for some kind of monitoring function. In such places, sensors harm may be a constant. For example, in a volcano- monitoring application, sensors may be destroyed by the expelled magma. After an earthquake, some buildings may become unstable, collapsing over deployed sensors after a while. Heavy rains may also damage deployed sensors. In all these cases, node failures may be unpredictable and may happen at any time during the network's expected operation lifetime.

Sensor nodes are powered by batteries and thus they have a finite energy supply. In fact, most sensors are powered by two AA batteries (3.3 V) with an estimated energy level around 20,000 J. If sensors run out of energy, they will go offline, unless some recharging or battery replacement mechanism is adopted. In short, energy recharging may be performed by harvesting energy from the environment [35], employing since solar panels or even unusual sources, such as tree movement [36]. Battery replacement is a bit more difficult, since sensor networks may be deployed over wide or even hard-to-access areas. Thus, for many networks, battery recharging will be unfeasible. On the other hand, many investigation efforts have been focused on energy-efficiency in wireless visual sensor networks, since the node lifetimes can be increased with a more efficient use of the available energy resources. Many works have addressed energy-efficiency in visual data transmission, congestion control, error recovery and packet routing, exploiting different characteristics for higher efficiency [2,3,8].

For visual monitoring performed by ordinary low-power cameras, luminosity will be required when retrieving visual information from the monitored field. Useless visual information may be retrieved when sensors are viewing regions with low (or absent) luminosity. For outdoor monitoring, sensors could become faulty near sunset and an unavailable network could be completely turned off for energy efficiency reasons.

Temporary or permanent interruption of communication links may also disconnect individual sensors or even a subnet. If some clusters fail or intermediate nodes closer to the sink run out of energy, the network may experience long periods of unavailability.

Finally, fabrication process problems may also affect the deployed sensors in different ways, whether related with the sensor basic hardware (processor, memory and energy supply) or the sensing unit. For visual sensors, the embedded camera brings other manufacturing problem possibilities, which may reduce the quality of the retrieved information or even turn it more fragile than usual or more power consuming.

Table 1 summarizes the most common hardware failure conditions for visual sensors. Once again, hardware failures affect sensors nodes whatever the monitoring requirements of the applications are, and thus their impact on the network availability is easier to evaluate.

Generally, a failure may be detected when disconnected nodes are identified or when the expected visual information never reaches the sink. Some works have focused on hardware failure detection, with different complexities [37]. In [38], the authors proposed a distributed faulty detection mechanism that analyzes neighbor data in order to identify disconnected nodes. In the same way, the work in [39] proposes a distributed failure detection mechanism, also addressing automatic recovery from hardware failures. Although relevant, those works are concerned with identification of connection losses. For wireless visual sensor networks, damages to the lens, for example, could inactivate a transmitting visual sensor, since it may then be considered as an unavailable node. Nevertheless, mechanisms to detect such kinds of failure are still lacking, demanding new investigation in the near future.

### 3.2. Coverage Failures

The directional sensing nature of visual sensors changes considerably the way information is gathered and processed by the applications, resulting in different perceptions of relevance and redundancy. In such a way, the impact of coverage failures on the network availability requires proper understanding of the application monitoring requirements, adding complexity to availability evaluation and enhancement.

The most trivial coverage failure is when visual data transmission ceases in the absence of hardware failures. Camera-enabled sensors may stop a current transmission according to their role in the application, due to a monitoring schedule or optimization mechanisms. In short, the application may decide to stop or reduce data transmission of a visual sensor as a mean of coverage optimization, directly producing a faulty condition. However, as the resulting faulty nodes were produced artificially, they may return to the normal transmission state at any time during the network operation.

Other possibility is the change of the monitoring requirements during the network lifetime. Such changes could alter the significance of all visual sources for the application, increasing or dramatically decreasing the availability level of the application. The proper perception of availability for this application may change considerably. As visual sensors may become useless for monitoring, we can consider this condition as a coverage failure.

**Figure 6.**
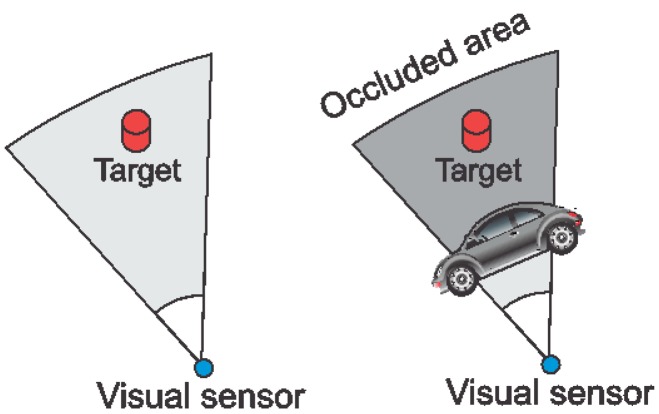
Occlusion in visual sensor networks.

When performing visual monitoring, the sensors FoV may be suddenly obstructed by some (mobile) obstacle. If such an obstruction is permanent, the visual sensor may become unavailable for the monitoring functions of the application. Occlusion is frequently unpredictable and may severely reduce the coverage area of one or more directional sensors, but the actual impact depends on the monitoring requirements. Figure 6 shows a graphical representation of occlusion, where a target is no longer viewed when an obstacle is located between it and the sensor's camera.

Besides occlusion, visual sensors may retrieve irrelevant information for the application if they are mobile. When moving across the monitored field (or beyond), the covered area will change with time and such changes may be hard to control or predict. In such a way, depending on the network configuration, some periods of unavailability may also be experienced.

A more complex situation is when sensors are retrieving visual information, but of low quality. This may happen due to some malfunction or a bad configuration. For example, the sensor's camera may be out of focus. Other possibility is when the camera lens is not clean, due to dust, fog or water. If infrared cameras are employed, regions with many heat sources may compromise an intrusion detection system. Those visual sensors may be considered faulty depending on the application monitoring requirements, which may indicate a quality threshold for the retrieved visual information. Table 2 summarizes the most common coverage failures for visual sensors.

It is interesting to note that we classified low-quality monitoring as a coverage failure, while low luminosity monitoring is a hardware failure. Once again, a sensor node with a hardware failure is assumed as unavailable for any type of application, while a coverage failure produces a faulty node depending on the application monitoring requirements. Visual data retrieved from dark regions will typically be considered as useless information for any application. Detection of coverage failures may be too hard to accomplish because different applications may have particular perceptions of monitoring quality and redundancy. Hence, availability evaluation must properly consider the particularities of the applications.

## 4. Availability Evaluation

We have discussed how redundancy should be considered to improve the availability level of wireless visual sensor networks. Moreover, we have presented some common hardware and coverage failures that can affect such availability level. However, when addressing availability in WVSNs, we should also consider mechanisms to evaluate the availability of these networks. We believe that three basics fundamentals aspects, namely redundancy, failure conditions and availability evaluation, are central when addressing availability in wireless visual sensor networks.

The availability level of WVSNs may be evaluated based on different aspects, such as node redundancy and connectivity [36–38,40], resulting in an expression of the network availability. However, this level can also be evaluated based on the potential of the deployed source nodes to provide useful and relevant information for the monitoring application. This particular perception is especially desirable for visual sensor networks, resulting in the concept of visual monitoring availability. In fact, visual availability is impacted in different levels by hardware failures and coverage failures, according to the way redundancy is defined for visual source nodes.

Mechanisms for availability evaluation are required when assessing the availability level of WVSNs. They may be used to indicate if the application has a satisfactory level of availability for the monitoring requirements or to anticipate decisions regarding network topology and deployment of redundant nodes. When a faulty operation is detected, the network can employ some mechanism to maintain the availability of the network at an acceptable level, but such level will vary considerably according to the characteristics of the monitoring application.

Visual sensor networks will be deployed to monitor a set of targets or scenes. The monitoring quality will then be associated with the capability of sensors to monitor some regions of the field. In this way, the effective visual coverage area of the network is of high importance. The deployed visual sensors will view part or the entire monitored field, which may be satisfactory or not for a particular monitoring application. As failures may reduce the area covered by visual nodes, redundancy will be exploited to compensate for the lack of visual coverage, according to the way source nodes are correlated (Section 2). In such way, we can expect that the monitoring requirements include a definition of targets or scenes to be monitored and the correlation of visual sensors when defining redundancy.

**Figure 7.**
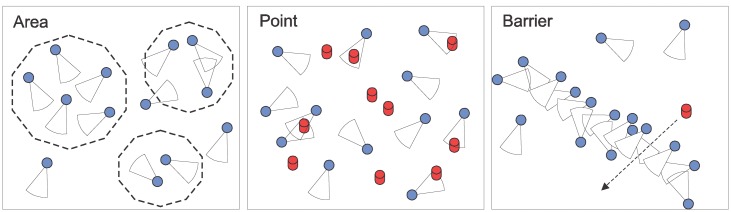
Visual monitoring by WVSNs.

Visual sensor networks may monitor the desired field in different ways. The work in [41] defines three types of coverage: area coverage, point coverage and barrier coverage. In area coverage, we are concerned with monitoring of one or more areas of the monitored field. The point coverage approach is focused on monitoring a set of targets. Finally, barrier coverage creates a conceptual barrier that avoids undetected penetration. Figure 7 presents a schema for these three types of visual monitoring requirements.

Typically, visual monitoring applications will have a minimum acceptable coverage of the monitored field, according to the nature of the monitoring application. Many applications may tolerate some node failures, since a minimum coverage level is still assured. This coverage level may be thought of as a quality threshold, where the network is assumed to be available as long as the minimum expected coverage level is respected. For example, a percentage of coverage of the monitored field may be defined as a minimum threshold for area coverage, whereas a minimum number of viewed targets could be defined for point coverage.

The coverage level of wireless visual sensor networks can be estimated considering some coverage metrics. In fact, coverage computation for visual sensors is more complex due to the nature of directional sensors and unpredictable problems such as occlusion and low luminosity, when compared with coverage estimation for traditional scalar sensors. Moreover, coverage failures are more critical for the monitoring functions of visual applications. In spite of that, we can employ some useful approaches to estimate the coverage level of the network, even partially.

The most trivial metric sums up the 2D or 3D occupation of the FoV of all visual sensors and divides the result by the area to be monitored, optionally using GPS information for better mapping of the coverage area. We can also simplify the FoV considering a triangular area, resulting in a FoV for each visual sensor equal to *α*^2^*r* (Figure 1). For a monitored field A, we can define ‖A‖ as the area to be monitored. In such way, each visual sensor will view *α*^2^*r*/‖*A*‖ of the monitored field. The final computed area can also be divided by the number of active nodes, resulting in an average result since sensor overlapping must be also considered. Although not precise, we can roughly estimate the level of coverage computing those FoV areas.

The area and point coverage paradigms are the most common approaches, but how the areas and targets are viewed depends on the way the camera's FoV will be processed. Thus, we could indicate that the network is available if and only if it can view 60% of the monitored field or 70% of the desired targets. Some nodes could become faulty, reducing the coverage area, but the network is assumed to be available as long as the minimum condition is still assured. Such restrictions could also define a minimum condition according to the initial configuration of the network just after deployment, even though such an initial configuration was not optimal.

For visual monitoring based on sensing relevance, a reasonable approach to implement a minimum acceptable availability level is considering the Quality of Viewing (QoV) concept [42]. QoV is a Quality of Experience (QoE) parameter [43] that is quantitatively measured to state the network monitoring quality. The QoV of a wireless visual sensor network is a 4-tuple of integers, {v_4_, v_3_, v_2_, v_1_}, representing the average measure of the quality for the source nodes assigned to the maximum relevance, high relevance, medium relevance and low relevance groups [21], respectively, as described in section 2.3. Each of these elements ranges from 0 (very low) to 100 (very high).

The QoV 4-tuple can be computed in two basic ways. The first option is to visually determine the level of satisfaction of the received data according to the associated group of relevance, where users will watch the received visual data and will rate the monitoring quality of each camera-enabled source node, and the average value will be considered for v_4_, v_3_, v_2_ and v_1_. The second way is by automatically computing the QoV 4-tuple, checking the coverage area. The visual monitoring quality can be computed measuring the level of visual coverage of the source nodes over the monitored field, according to predefined areas of interest. In Figure 5, the QoV could indicate the average percentage of coverage of the visual sensors over the defined squares of relevance.

The computed QoV level can be used to indicate a minimum acceptable coverage quality for the network, which in turn may be perceived as a minimum acceptable availability level. For example, one particular application may define that the network is available while the QoV is equal or higher than {100, 80, 50, 0}. In such way, if QoV = {100, 90, 40, 100}, the network is assumed to be unavailable, since the value for v_2_, 40, is less than 50, the minimum acceptable level. The QoV value can then be used to trigger some mechanism to reestablish the minimum desired level of coverage (e.g., by activating redundant sleeping nodes), trying to turn the network available again.

In barrier monitoring, the availability may be evaluated as the capacity of the visual sensor network to view targets that cross a predefined conceptual line. In fact, it is a very useful approach for intrusion detection. The idea is that sensors need to be deployed to cover only a path that completely crosses the width or height of the monitored field, in order to detect the crossing of a moving object [44]. When planning the expected level of availability, we can define that the network is available as long as the barrier is maintained, even if some visual sensors become faulty.

Barrier monitoring can be classified in *weak* and *strong* [44]. In weak barrier coverage, sensors must detect an object that is moving along congruent paths, while strong barrier coverage would detect any type of movement behavior. When estimating availability, we may be most concerned with strong barrier coverage for more accurate detection of unauthorized penetration.

Some coverage metrics for scalar sensor networks can contribute to the availability evaluation of WVSNs. The work in [45] investigates coverage in scalar wireless sensor networks, classifying the achieved coverage in three different categories: full coverage with connectivity, partial coverage with connectivity and coverage with constrained connectivity. The author argues that coverage without connectivity is meaningless in wireless sensor networks, resulting in disconnected subnets, and the same idea may be valid for wireless visual sensor networks. The work in [22] defines the *K*-Coverage metric, which says that every point in the deployed region is within the coverage ranges of at least *K* source nodes. Based on this concept, the Directional *K*-Coverage (DKC) metric is proposed in [46], adapting the concept previously defined in [22] in order to consider directional visual monitoring. DKC is defined as a probability guarantee, since 100% coverage is very difficult to achieve for randomly deployed visual sensors with a uniform density [46].

There are also some valuable metrics for computation of the visual coverage of camera-enabled sensors. The work in [47] proposed a polynomial time algorithm to compute worst-case coverage, which is related with the maximal distance that a mobile target can maintain from the sensors. In [48] the Deployment Coverage Quality metric is proposed, as a ratio between the sum of all relevant sensing areas and the network area considering directional coverage. In [49] several coverage algorithms are compared and a new distributed algorithm is proposed to optimize coverage. Some valuable metrics are also discussed in [50]. All these metrics are relevant to indicate the visual quality of monitoring applications, indirectly assessing the availability level, but we can expect that metrics that can compute the coverage area based on some particularities as target faces and desired orientation of monitoring will be more appropriate when assessing the availability level, whenever only a single perspective of the targets is desired.

Besides computation of coverage metrics, current monitoring quality could be evaluated considering the perception of the network by its users. In such way, retrieved visual information could be watched by users who would rate it according to the expected information when planning the network. Based on the final ratings the network could be assumed available or not, although such approach may suffer from unconscious psychological factors [43]. Table 3 summarizes the discussed metrics for availability evaluation in wireless visual sensor networks.

The coverage estimation of wireless visual sensor networks may provide some numerical indication that can reflect an availability level, which may be assumed as acceptable or not according to the application monitoring requirements. Hardware and coverage failures may reduce the coverage area of the network when fewer or lower quality visual data are retrieved, but redundancy may replace the faulty nodes. Together, the particular perception of redundancy of visual sensors (Section 2), the hardware and coverage failures (Section 3) and the availability evaluation based on visual coverage (Section 4) provide the basic concepts that are required when addressing availability in wireless visual sensor networks.

## 5. Enhancing Availability

The evaluation of the availability level of visual monitoring applications is a generally desired property. Knowing the possible hardware and coverage failures, the particularities of redundancy and the minimum acceptable availability of the visual monitoring application, as well as its current availability level, it is possible to assess the overall quality of the wireless visual sensor network, indicating if a particular application is being currently adequate for its expected monitoring functions. But when a monitoring application is assumed as unavailable, some mechanisms can be applied to increase its availability. We expect that applications availability can be enhanced through at least four different approaches: optimized deployment, coverage optimization, increasing redundancy and energy efficiency. We discuss these approaches in the next sections.

### 5.1. Optimizing Deployment

Monitoring applications may have different requirements that must be properly considered, indicating how the network should be deployed. For example, a WVSN may be deployed for monitoring a previously known set of targets (point coverage). In a different way, monitoring functions may be expected to be performed in delimited areas (area coverage), where targets may be already present or expected to cross (barrier coverage). Moreover, some applications may not require 100% coverage of the desired targets or areas, requiring, for example, only 70% of coverage over the monitored field. Other relevant requirements are related with the way information will be retrieved (clock-based, query-based or event-based transmission [5]), which may guide the formation of the network topology. Finally, some applications may expect that heterogeneous nodes will be deployed, where some nodes may have better resources for sensing and computing, besides more powerful energy supplies. All these characteristics might impact the way sensors are deployed, directly affecting the network availability.

Typically, wireless sensor networks may be randomly or deterministically deployed. In random deployment, sensors will be scattered over a target area, what may result in some regions being densely or sparsely covered by visual sensor nodes. Sensors may be airdropped, launched in rockets or released over the ocean, just to cite some approaches, depending on the application monitoring requirements and the characteristics of the area to be monitored. Despite the inner complexity resulting from the lack of exact positioning of nodes, the employed random deployment mechanism may indirectly result in damage to camera-enabled sensors, creating a failure condition. Nevertheless, it may be the only feasible approach for deployment in harsh and hard-to-access environments. On the other hand, sensors may be deterministically placed following a pre-processed plan. Such an approach allows the covered area to be maximized with a minimum number of sensors, potentially allowing efficient planning of redundant nodes for higher availability.

In random deployment, the resulting node locations and camera orientations cannot be predicted. In order to compensate for such limitations, nodes are typically deployed in excess [46,51]. Considering visual monitoring based on similarity, the deployed network may become unavailable even before the beginning of the sensing monitoring functions, in the case the desired targets are not viewed (or only partially viewed). In such case, if the target locations are known before deployment, sensors could be airdropped closer to them, although their landing position cannot be precisely predicted. The work in [52] proposes a deployment algorithm that uses a configuration file containing information about the area to be monitored, such as its length and width, the location of the sink and the obstacles (that may generate occlusions). We could also add information about the desired targets to be monitored, creating a more complete configuration file for the optimization process. For visual monitoring based on overlapping, we could face the same coverage maximization problem, with the difference that redundant nodes could be easier to identify. Finally, for visual monitoring based on sensing relevance, we could use information about the regions of interest in order to guide the deployment process. In regions of interest with higher relevance for the application, more sensors or sensors with better resources could be deployed to enhance the overall monitoring quality and consequently the application availability.

Sometimes, visual sensors can be deployed deterministically. Deterministic positioning may be highly beneficial for a large set of monitoring applications, since the covered area may be optimized with high accordance with the applications' monitoring requirements. Many works have proposed different algorithms for optimal camera placement [9,14], where it is desired to find the minimal number of nodes that can view a larger area of the monitored field. Concerning availability, maximum deployment with an optimal number of visual sensors can generate more inactive redundant nodes that can be exploited to replace faulty nodes, making the network available for longer. However, such optimizations are only valid depending on the way the sensors' FoV are interpreted by the application.

As a final comment, it should be considered that for areas with high occlusion, many low-resolution visual sensors are a much better solution than a few high-resolution cameras [53–55], and increasing the deployment density expands the coverage area, but not in the same proportion [9]. Thus, a massive deployment of low-cost visual sensors will be frequently a better approach for any type of visual monitoring.

### 5.2. Coverage Optimization

As the availability level of monitoring applications can be inferred from the coverage area of the deployed visual sensors, the effective FoV of those sensors can be optimized to enhance the application availability. Sometimes, cameras with adjustable FoV or even mobile sensors may be deployed, allowing some level of coverage optimization. Although some algorithms may try to optimize the sensor deployment [9], as discussed earlier, the final configuration after deployment may be sub-optimal for many reasons.

After deployment, some desired targets may be not viewed by the visual sensors, even for a high number of deployed nodes. If so, availability evaluation based on coverage of those targets may indicate that the network is unavailable, even before any visual data transmission is requested. Many works have proposed different algorithms to address camera calibration for optimal coverage of the monitored field [9,20,50], but generally this is an NP-hard problem.

The work in [51] presents two centralized and one distributed algorithm to compute the optimal orientations of visual sensors in order to cover as many targets as possible. Doing so, the minimum number of sensors is activated, enhancing availability with a better coverage of the monitored field and also providing more redundant nodes. The orientations computed in [51] are used to change the directions of active visual sensors. It is also showed that by increasing linearly the number of deployed sensors the coverage ratio and the number of active nodes are increased, until the deployed sensors reach a threshold. The authors of [56] also propose algorithms to indicate the orientations that each sensor has to assume to optimally cover all targets. The directions of the sensors are organized into non-disjoint subsets, allowing sensors to participate in multiple sets. Such cover sets could be mapped to groups of relevance, allowing optimizations according to the regions defined for each group.

In general terms, any FoV optimization algorithm must consider the way visual information is gathered from the monitored field and how redundancy is defined, as discussed in Section 2. The monitoring requirements will indicate how visual sensors should view targets or scenes, and this information should be an input in the coverage optimization process.

When optimizing coverage, we may want to reduce occlusion. Visual sensors with occluded FoV may impact the evaluated availability level of the application. If the orientation of the deployed sensors can be changed, the FoV can be adjusted to view useful areas of the monitored field. In [53] a distributed method to change the orientation of visual sensors is proposed, aimed at reducing the effects of occlusion. In the proposed procedure, each node independently discovers its neighbors and analyzes obstacles and overlapping areas. According to the information discovered from the neighborhood, nodes can automatically adjust their orientations, optimizing the coverage area.

### 5.3. Increasing Redundancy

Redundancy is a feasible way to enhance the availability level of wireless visual sensor networks and their applications. In fact, redundant nodes may be created after massive deployment or when coverage optimization algorithms are employed, but different applications may define different redundant nodes for the same network, depending on the visual monitoring requirements.

The first step to enhance availability through redundancy is identifying redundant nodes. For that, the easier approach is discovering if sensors can view the same target. For example, a particular monitoring application may define that two sensors have equivalent monitoring if they have at least 60% of FoV overlap and thus one of them could be assumed as redundant. On the other hand, a different application could define that 80% of overlapping is required when defining redundancy. Moreover, the angle between the cameras' orientations may also be considered, as defined in Section 2. In fact, there is no universal formulation for what is equivalent monitoring, since it depends on the application monitoring requirements.

Some recent works have addressed redundancy in wireless visual sensor networks. As said before, the work in [51] presents centralized and distributed algorithms to control the number of active source nodes needed for optimal visual coverage. Redundant nodes are turned off to preserve energy, potentially prolonging the network lifetime. For that, the optimized positioning of visual sensors is computed, activating the minimum number of sensors, as also proposed in [57]. Another relevant contribution is that of [57] where active sensors that are closer to the sink are selected since packets transmitted from them will have to cross fewer links toward the destination. The work in [58] proposes a metric to identify less relevant redundant nodes, where relevance is a function of overlapped areas. For all these approaches, algorithms should be optimized to reflect the notion of redundancy expected for the considered application.

In scalar wireless sensor networks, the *K*-Coverage metric indicates that every point in the deployed region is within the sensing ranges of at least *K* sensors [22]. For example, in a *3*-Coverage network, a failure of one or two nodes sensing the same region still maintains that particular region covered, but not necessarily connected. In [59] it is showed that if the communication range is at least twice the sensing range, a *K*-Covered network results in a *K*-Connected network. Although relevant, the *K*-Coverage concept cannot be directly considered in WVSNs, since in most cases the sensing range of visual sensors is directional (ominidirecional cameras may be also deployed).

The work in [46] considers the directional sensing nature of visual sensors when dealing with the *K*-Coverage problem. In that work the Directional *K*-Coverage metric is proposed, which estimates the probability that all points in the monitored field are viewed by *K* sensors. The authors in [46] argue that for a random deployment with uniform density, it is difficult (if not impossible) to guarantee 100% of directional *K*-Coverage. Thus, the idea is to estimate the probability to achieve a directional *K*-Coverage configuration, according to the number of deployed visual sensors. The DKC metric was defined in [46] to evaluate the number of visual sensors that can view the “face” of the target (visual monitoring based on similarity). For example, in an intrusion detection system, it may be required that visual sensors can view people's faces, supporting facial recognition mechanisms. For that, a target to be viewed must be inside the FoV of the camera and the orientation angle of the face must be also viewed. Note that the authors do not propose DKC to estimate redundancy, but as a mechanism to estimate the probability that the face of targets can be viewed by *K* sensors, even in different perspectives, since some detection algorithms may require different viewings of the face of targets. However, this notion could be also employed to estimate the redundancy probability for visual monitoring after random deployment.

When employing the DKC metric, we could identify that the network has, for example, a probability of 70% to be in D*3*-Coverage and an 85% probability to be in D*2*-Coverage. Of course, this depends on the dimensions of the monitored field, the targets' configurations and the number of deployed nodes, but it was concluded in [46] that higher values for DKC will be achieved when more visual sensor nodes are deployed. In such way, the availability requirements could be considered when defining the appropriate number of visual sensors to deploy.

The DKC metric may then indicate a probability that a network will have redundant nodes after a random uniform deployment for visual sensors with close orientations, directly benefiting visual monitoring based on similarity. However, we could extend the DKC metric to consider 360° face angles, indicating that an effective coverage is achieved only by considering FoV overlap and the positions of the targets. In such a way, we could use this metric for visual monitoring based on overlapping, potentially increasing the probability of redundancy after deployment.

The formulation in [46] does not consider the border effect. The border effect results from the fact that sensor nodes near the border of the monitoring field may cover a lesser area than sensors placed midway [60]. For scalar sensors, the border effect has a deeper impact because the sensing area is omnidirectional. However, for directional sensing, visual sensors may be positioned on the border of the target area and their FoV may be completely inside the monitored field, or the opposite. In [60] a theoretical and experimental analysis of the border effect is conducted for scalar sensor networks, achieving more realist results when computing *K*-Coverage. The same idea may apply for directional sensors when computing DKC, but this requires proper consideration of the particularities of visual sensing.

**Figure 8.**
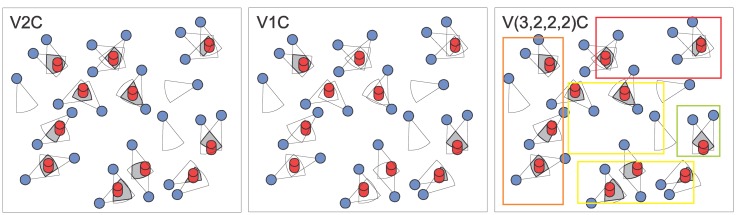
Visual *K*-Coverage metric according to visual redundancy.

We propose the Visual *K*-Coverage (VKC) metric as a guarantee that every target (from a known set of targets) is viewed by at least *K* visual sensors and that those sensors can be assumed as redundant. The VKC metric must be computed according to the application monitoring requirements, which must indicate the parameters that define redundancy: FoV overlap, cameras' orientations and areas of interest. For this last case, the proposed metric could also indicate the value of *K* for each group of relevance, achieving the value of V(*K_4_*,*K_3_*,*K_2_*,*K_1_*)C, respectively standing for the maximum, high, medium and low relevance groups, from *K_4_* to *K_1_*. Figure 8 presents an example of a wireless visual sensor network with different values for the Visual *K*-Coverage metric. The differences result from the way redundant nodes are defined. The color patterns for the areas of interest are the same employed in Figure 5.

As already defined, the Visual *K*-Coverage metric depends on the way redundancy is considered. The WVSN in Figure 8 has V2C when redundancy is only based on overlapping. However, when visual redundancy is based on similarity, the network becomes V1C. Finally, FoV overlapping was assumed when computing the V(*K_4_*,*K_3_*,*K_2_*,*K_1_*)C, but if redundancy based on similarity was considered along with the sensing relevancies of source nodes, the network could become V(1,1,1,2)C, depending on the minimal FoV overlapping area and the angle between cameras' orientations.

When visual sensors are assigned to different sensing relevancies according to the application monitoring requirements, a level of redundancy could be computed for every area of interest, according to the number of deployed visual sensors that can view the defined areas. By doing so, we could define redundant nodes inside each area, what could lead us to perceive different levels of availability for the same network.

We could also find relevant redundancy issues for barrier coverage in wireless visual sensor networks. The work in [61] addresses strong barrier coverage by visual sensors. In fact, barrier coverage becomes more complex when employing directional sensors when compared with ominidirecional sensors. In [61] an algorithm is proposed to find the minimal set of visual sensors that creates a barrier-like coverage area. When concerning the network availability, we could expect that the network is available as long as the barrier is preserved. In such way, redundant nodes could be employed to replace faulty nodes or new nodes could be deployed to compensate for the uncovered areas. However, we can further extend the availability level of the network by creating multiple levels of barriers. For example, in an intrusion detection system, visual sensors could be deployed in such way that two disjoint barriers would be composed. The created 2-Barrier WVSN would provide a higher level of availability, since a mobile target would not be detected only if it could move across both barriers without being viewed by any visual sensor.

We expect that *B* disjoint visual barriers can be created in wireless visual sensor networks. For this *B*-Barrier coverage problem, we assume that a particular barrier becomes unavailable when a coverage hole is created due to a hardware or coverage failure of one or more visual sensors. If we assume *f* as the (homogeneous) probability for the creation of a single hole in any barrier, the unavailability probability would be *f^B^*. In other words, for higher *B*, it would be more difficult that a mobile target would move undetected through all barriers.

An efficient *B*-Barrier visual sensor network would be created with careful deployment of camera-enabled nodes. Many works have been concerned with the way visual sensors will be deployed and how the coverage area may be optimized to reduce the number of active nodes, as discussed before. In such way, higher availability is expected from deterministic deployment in most cases [9], where *K*-Coverage or *B*-Barrier configuration could be more easily achieved. The work in [61] investigates optimal coverage of visual sensors when they are deterministically deployed, considering the connectivity of the sensors as a relevant aspect to be preserved and optimized. A similar investigation is conducted in [14]. A deterministic deployment could indeed be used to create a broader concept of Visual *K*-Coverage, where a Visual *3*-Coverage, for example, would indicate a high probability that all points in an area (not only predefined targets) are viewed by three visual sensors, where availability requirements would be related with the definition of such high probability threshold.

### 5.4. Energy Efficiency

Wireless visual sensors networks will be typically composed of battery-operated nodes where the energy supply is finite. When the energy resources are depleted, sensor nodes or segments of the network may become inactive. In order to reduce energy consumption when retrieving and transmitting visual information, many works have proposed energy-efficient optimizations, bringing significant results for availability enhancement in these networks.

Visual sensors will transmit still images or video streams to the network sink. Generally, visual information will require more bits than scalar information like humidity, pressure and temperature, making visual data transmission more stringent. In such way, we can expect that the number of active visual sensors will be related with the network lifetime and thus the way information is transmitted is a relevant availability issue.

Due to the energy constraints of sensor nodes, communication protocols must be energy-efficient and the transmission power of the employed radio hardware must be low. When dealing with visual sensors, low-power cameras should be embedded for higher efficiency.

A crucial aspect of energy-efficiency is visual data coding [8]. Due to the expected resource constraints of the visual sensors, codecs should be simple enough to consume low energy. However, high compression is desired to reduce the amount of information to be transmitted over the network. This tradeoff has fostered the development of optimization mechanisms that discard lower relevant information in order to reduce energy consumption [2,4,8]. Other research line is focused on Distributed Video Coding, where the encoders are less complex than the decoders and thus the complexity is shifted to the sink, which is expected to be resource-full [8,62].

Besides data coding, wireless visual sensor networks will have to deal with other relevant energy-demanding issues. As visual sensors may transmit huge amounts of information, when compared with traditional scalar sensor networks, the transmission paths may be congested. In short, congested nodes may prejudice the overall monitoring quality of the application and more energy may be consumed when lost packets need to be retransmitted. As wireless *ad hoc* communications are expected to be error-prone, packet corruption may also impact the energy resources of the nodes. In order to deal with such complex environment, many works have proposed different optimizations for energy efficiency, with different results in terms of energy preservation and visual monitoring quality [1,2,8].

Although relevant, energy preservation and the related network optimizations should be applied in different ways, according to the monitoring requirements. For example, if FoV overlap is considered when defining redundancy, energy preservation of visual sensors may not be so crucial (in some cases) when compared with monitoring based on similarity. From the same perspective, if visual sensors have different sensing relevance, the energy resources of higher relevant sensors are more critical and optimization mechanisms should be more concerned with those sensors. In fact, if we are estimating the availability level of the application by the effective visual monitoring capability, energy-efficiency must be thought through considering the particularities of each type of monitoring.

## 6. Conclusions

Wireless visual sensor networks are a valuable resource for many surveillance, tracking and general-purpose monitoring applications. Camera-enabled sensors will retrieve visual information that can be exploited for public security, military surveillance, industrial automation, weather monitoring, rescue operations, traffic management uses, among many others. For some of those applications, however, there will be some criticality in the performed monitoring, requiring high availability throughout the network lifetime. For example, in a fire prevention and control system, a neglected failure of a visual sensor may compromise the effectiveness of the sensor network. In such way, the network availability becomes a relevant design issue.

Availability is highly relevant for modern wireless visual sensor networks, but it has not been properly investigated. The availability concepts for wireless sensor networks are not suitable for WVSNs, since visual sensors collect visual information following a directional sensing model and source nodes may have different relevancies for the applications. The notion of redundancy is also different, requiring a new understanding of faulty node replacement. In this work we identified the main availability issues in wireless visual sensor networks, discussing mechanisms to evaluate and enhance their availability level.

The high complexity of wireless visual sensor networks requires a deeper understanding of key concepts of these networks, prompting us to consider a particular perception of availability. In this paper we have discussed many relevant issues that must be properly considered in order to assure acceptable levels of availability for WVSNs. We believe that the covered topics may help in new research lines in this area.

## Figures and Tables

**Table 1.** Most common hardware failures in wireless visual sensor networks.

**Failure Condition**	**Impact in WVSNs**
Bad deployment	Embedded cameras may be damaged during deployment. Deployed cameras may have suboptimal FoV and/or view undesired areas.
Damages in harsh areas	Sensor nodes may be harmed, becoming unavailable.
Energy depletion	Energy is more critical for camera-enabled sensors, since transmission of visual data is more stringent than transmission of scalar data.
Low luminosity	Visual sensors may be unable to retrieve useful images or videos from regions with low luminosity.
Connectivity loss	Sensor nodes may go offline if there is no active transmission path to the network sink.
Fabrication process	Problems during fabrication may result in different failures that may happen at any time along the network operation, including problems during visual monitoring.

**Table 2.** Most common coverage failures in wireless visual sensor networks.

**Failure Condition**	**Impact in WVSNs**
Coverage optimization	Faulty nodes are artificially produced due to a monitoring schedule or coverage optimization. The set of nodes selected depends on the way visual information is monitored by the application.
New monitoring requirements	Changing the monitoring requirements may alter the role of the visual sensor for the application.
Occlusion	Desired targets may not be viewed due to occlusion, making the affected source node unavailable.
Low-relevance monitoring	Mobile nodes may retrieve information of low relevance for the application. This may also happen with static sensors with dynamic FoV adjustment.
Low-quality monitoring	Environmental conditions or bad configuration and adjustment of the sensors' cameras may reduce the quality of the retrieved visual information.

**Table 3.** Availability evaluation in WVSNs.

**Metric**	**Description**
Coverage quality [47–50]	The availability level is a function of the area covered by visual sensors. Applications may define a minimum threshold for the area covered by all visual source nodes.
Quality of Viewing [42]	Coverage is defined for groups of relevance and thus the availability level of the network depends on the way visual sources retrieve information from each group of relevance.
Barrier monitoring [44]	The network is assumed available as long as the conceptual barrier is maintained.
Directional *K*-Coverage [46]	Probability of the visual sensor network to be *K*-Coverage.
Users perceptions	The availability level of the network is indirectly inferred from the perception of the users over the retrieved visual data.
